# Mechanisms underlying divergent responses of genetically distinct macrophages to IL-4

**DOI:** 10.1126/sciadv.abf9808

**Published:** 2021-06-16

**Authors:** Marten A. Hoeksema, Zeyang Shen, Inge R. Holtman, An Zheng, Nathan J. Spann, Isidoro Cobo, Melissa Gymrek, Christopher K. Glass

**Affiliations:** 1 Department of Cellular and Molecular Medicine, School of Medicine, University of California, San Diego, La Jolla, CA 92093, USA.; 2 Department of Bioengineering, Jacobs School of Engineering, University of California, San Diego, La Jolla, CA 92093, USA.; 3 Section Molecular Neurobiology, Department of Biomedical Sciences of Cells and Systems, University Medical Center Groningen, University of Groningen, Groningen, Netherlands.; 4 Department of Computer Science and Engineering, University of California, San Diego, La Jolla, CA 92093, USA.; 5 Department of Medicine, School of Medicine, University of California, San Diego, La Jolla, CA 92093, USA.

## Abstract

Mechanisms by which noncoding genetic variation influences gene expression remain only partially understood but are considered to be major determinants of phenotypic diversity and disease risk. Here, we evaluated effects of >50 million single-nucleotide polymorphisms and short insertions/deletions provided by five inbred strains of mice on the responses of macrophages to interleukin-4 (IL-4), a cytokine that plays pleiotropic roles in immunity and tissue homeostasis. Of >600 genes induced >2-fold by IL-4 across the five strains, only 26 genes reached this threshold in all strains. By applying deep learning and motif mutation analyses to epigenetic data for macrophages from each strain, we identified the dominant combinations of lineage-determining and signal-dependent transcription factors driving IL-4 enhancer activation. These studies further revealed mechanisms by which noncoding genetic variation influences absolute levels of enhancer activity and their dynamic responses to IL-4, thereby contributing to strain-differential patterns of gene expression and phenotypic diversity.

## Teaser

Genetic variants in transcription factor motifs result in divergent IL-4 responses in macrophages from inbred strains of mice.

## INTRODUCTION

Noncoding genetic variation is a major driver of phenotypic diversity and the risk of a broad spectrum of diseases. For example, of the common single-nucleotide polymorphisms and short insertions/deletions identified by genome-wide association studies to be linked to specific traits or diseases, ~90% are typically found to reside in noncoding regions of the genome (*1*). The recent application of genome-wide approaches to define the regulatory landscapes of many different cell types and tissues allows intersection of these variants with cell-specific regulatory elements and strongly supports the concept that alteration of transcription factor binding sites at these locations is an important mechanism by which they influence gene expression (*2*–*4*). Despite these major advances, it remains difficult to predict the consequences of most forms of noncoding genetic variation. Major challenges that remain include defining the causal variant within a block of variants that are in high linkage disequilibrium, identifying the gene that is regulated by the causal variant, and understanding the cell type and cell state–specific regulatory landscape in which a variant might have a functional consequence (*5*). For example, a variant that affects the binding of a signal-dependent transcription factor (SDTF) may only be of functional importance in a cell that is responding to a signal that activates that factor (*6*). Also, sequence variants can have a range of effects on transcription factor binding motifs, from abolishing or inducing binding by affecting critical nucleotides to quantitatively changing binding by affecting an intermediate affinity motif (*7*–*9*).

Studies of the impact of natural genetic variation on signal-dependent gene expression have demonstrated large differences in absolute levels of gene expression under basal and stimulated conditions, which result in corresponding differences in the dynamic range of the response (*10*–*12*). The molecular mechanisms by which genetic variation results in these qualitatively and quantitatively different signal-dependent responses remain poorly understood but are likely to be of broad relevance to understanding how noncoding variation influences responses to signals that regulate development, homeostasis, and disease-associated patterns of gene expression.

**Fig. 1:**
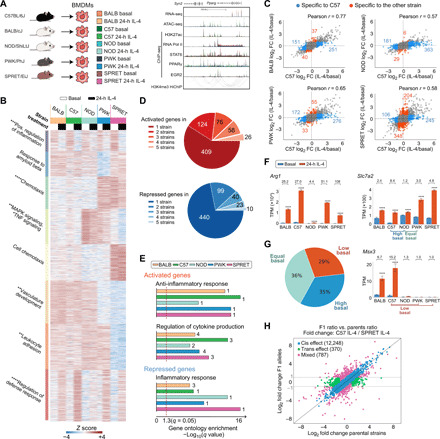
Response to IL-4 is highly divergent in bone marrow–derived macrophages from different mouse strains. (**A**) Overview of experimental design and main datasets. (**B**) WGCNA clustering focused on strain-differentially regulated genes in IL-4–treated bone marrow–derived macrophages (BMDMs). The top hit Metascape pathways are annotated for each module. *q < 0.05, **q < 0.01, ***q < 0.001, ****q < 0.0001. MAPK, mitogen-activated protein kinase; TNF, tumor necrosis factor. (**C**) Ratio-ratio plots demonstrating the mRNA response to IL-4 in pairwise comparisons. (**D**) Overlap of genes significantly induced or repressed (*q* < 0.05, >2-fold) after IL-4 treatment in BMDMs from all strains. (**E**) Gene ontology terms enriched in up- and down-regulated genes after 24-hour IL-4 stimulation in BMDMs from all strains. Numbers indicate the rank order in pathway analysis. (**F**) *Arg1*, *Slc7a2*, and *Msx3* as example genes differentially up-regulated by IL-4 in strains. TPM, transcripts per kilobase million. ****q < 0.0001, compared to basal. Numbers indicate fold change by IL-4. (**G**) Categories of strain-differential IL-4 up-regulated genes based on the differences in basal gene expression. (**H**) Average log_2_ gene expression fold change between alleles in hybrid (C57 × SPRET F1) and parental strain under 24-hour IL-4 conditions.

To investigate the influence of genetic variation on signal-dependent gene expression, we performed transcriptomic and epigenetic studies of the responses of macrophages derived from five different inbred mouse strains to the anti-inflammatory cytokine interleukin-4 (IL-4) (Fig. 1A). The selected strains include both similar and highly divergent strain pairs, allowing modeling of the degree of variation between two unrelated individuals (~4 million variants) and that observed across large human populations (>50 million variants). Using this approach, we previously showed that strain-specific variants that disrupt the recognition motif for one macrophage lineage-determining transcription factor (LDTF; e.g., PU.1), besides reducing binding of the LDTF itself, also result in decreased binding of other collaborative factors and SDTFs (*13*, *14*). Collectively, these findings supported a model in which relatively simple combinations of LDTFs collaborate with an ensemble of additional transcription factors to select cell-specific enhancers that provide sites of action of broadly expressed SDTFs (*15*).

IL-4 has many biological roles, including regulation of innate and adaptive immunity (*16*). In macrophages, IL-4 drives an “alternatively activated” program of gene expression associated with inhibition of inflammatory responses and promotion of wound repair (*17*). The immediate transcriptional response to IL-4 is mediated by activation of signal transducer and activator of transcription 6 (STAT6) (*18*, *19*), which rapidly induces the expression of direct target genes that include effector proteins such as Arginase 1 (Arg1) and transcription factors like peroxisome proliferator–activated receptor γ (PPARγ) (*20*, *21*) and early growth response 2 (EGR2) (*22*). However, the extent to which natural genetic variation influences the program of alternative macrophage activation has not been systematically evaluated. Here, we demonstrate highly differential IL-4–induced gene expression and enhancer activation in bone marrow–derived macrophages (BMDMs) across the five mouse strains, thereby establishing a robust model system for quantitative analysis of the effects of natural genetic variation on signal-dependent gene expression. Through the application of deep learning methods and motif mutation analysis of strain-differential IL-4–activated enhancers, we provide functional evidence for a dominant set of LDTFs and SDTFs required for late IL-4 enhancer activation, which include STAT6, PPARγ, and EGR2, and validate these findings in *Egr2*-knockout BMDMs. Assessment of the quantitative effects of natural genetic variants on recognition motifs for LDTFs and SDTFs suggests general principles by which such variation affects enhancer activity patterns and dynamic signal responses.

## RESULTS

### The response to IL-4 is highly variable in BMDMs from genetically diverse mice

To investigate how natural genetic variation affects the macrophage response to IL-4, we began by performing RNA sequencing (RNA-seq) in BMDMs derived from female BALB/cJ (BALB), C57BL/6J (C57), NOD/ShiLtJ (NOD), PWK/PhJ (PWK), and SPRET/EiJ (SPRET) mice under basal conditions and following stimulation with IL-4. Time-course experiments in C57 BMDMs indicated a progressive increase in the number of differentially expressed genes from 1 to 24 hours (fig. S1, A and B, and table S1). We therefore focused our analysis on the response to IL-4 in BMDMs from the five strains at this time point. Weighted gene coexpression network analysis (WGCNA) identified numerous modules of highly correlated mRNAs, most of which were driven by strain differences (Fig. 1B). Genes that were positively regulated by IL-4 across strains (red module, bottom) were enriched for functional annotations related to negative regulation of defense responses. Conversely, the purple (top) module captured genes that were negatively regulated by IL-4 and were enriched for pathways associated with positive regulation of inflammation (Fig. 1B).

Of the 693 genes induced >2-fold in at least one strain, only 26 (3.75%) were induced at this threshold in all five strains (Fig. 1D, fig. S1, C and D, and table S2). Conversely, more than half of the IL-4–responsive genes identified were induced >2-fold in only a single strain. NOD BMDMs were notable for a generally attenuated response to IL-4 (Fig. 1B, red module, and Fig. 1C, second panel). A similar pattern was observed for down-regulated genes (Fig. 1D). Despite these differences at the level of individual genes, similar pathways/gene programs were enriched in all strains for both induced and repressed genes (Fig. 1E). Substantial differences in IL-4 target gene expression across strains are illustrated by *Arg1*, *Slc7a2*, and *Msx3* (Fig. 1F and fig. S1E). BMDMs from all strains exhibit a significant induction of *Arg1* expression, but the absolute basal levels and induction folds vary by more than an order of magnitude. *Slc7a2* exhibits similar levels of expression in C57 and NOD BMDMs after IL-4 treatment, but its differences at the basal level result in an 8.4-fold and 1.2-fold change, respectively. We refer to the pattern of reduced responsiveness to IL-4 in this comparison of C57 and NOD as being associated with “high basal” activity in the less responsive strain. Conversely, NOD and PWK BMDMs exhibit similar levels of basal *Slc7a2* expression, but IL-4 only increased *Slc7a2* expression more than twofold in PWK. We refer to this pattern of reduced responsiveness to IL-4 in NOD compared to PWK as being associated with “equal basal” activity. A third category is exemplified by *Msx3*, which is induced in C57 but not in PWK and SPRET BMDMs. In this case, lack of responsiveness is associated with low expression of *Msx3* under basal conditions. We refer to this pattern as “low basal” in the less responsive strain. Quantitative analyses of pairwise comparisons indicate that 29% of the genes with decreased IL-4–induced gene expression were due to low basal expression, 36% had no differences before IL-4 stimulation (equal basal), and 35% were the result of a high basal expression level in the less responsive strain (Fig. 1G).

To investigate local versus distant effects of genetic variation on the differential responses to IL-4, we crossed C57 mice with the most genetically distinct SPRET mice to generate F1 offspring containing each parental chromosome. A total of 91.4% of parental-specific RNA-seq reads in the F1 strain are within twofold of their values in C57 and SPRET (blue data points) and considered to be due to local (cis) effects of genetic variation (Fig. 1H and fig. S1F), while only 2.8% were divergent between the parental strains but not in F1 BMDMs (green data points), indicating trans regulation. As NOD macrophages exhibited a broadly attenuated response to IL-4 on the level of gene expression, we followed the same strategy using F1 C57 × NOD macrophages. RNA-seq on IL-4–stimulated macrophages of F1 C57 × NOD macrophages showed strong convergence of expression of genes that were differentially regulated in the parental strain (green data points in fig. S1G), consistently a major contribution of trans regulation. To investigate the point at which this regulation occurs, we performed chromatin immunoprecipitation sequencing (ChIP-seq) for RNA polymerase II (Pol II) under control and IL-4–stimulated conditions. In contrast to mRNA levels, examination of the IL-4–dependent changes in gene body RNA Pol II indicated similar magnitude changes in all strains, including NOD (fig. S1H). These results suggest the presence of a transacting factor in NOD that acts downstream of transcription to attenuate mRNA levels. Collectively, these studies uncovered notable variation in the cell autonomous responses of BMDMs to IL-4 across these five strains, providing a powerful experimental system for investigating mechanisms by which natural genetic variation affects signal-dependent gene expression.

### Strain-differential IL-4–induced gene expression is associated with differential IL-4 enhancer activation

**Fig. 2:**
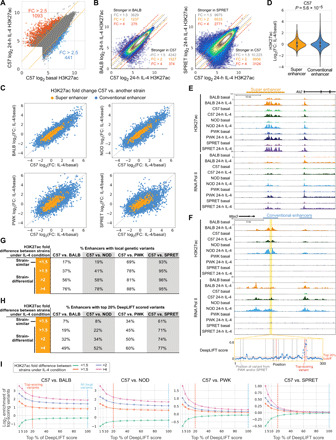
Divergent IL-4 response is associated with strain-differential IL-4 enhancer activation. (**A**) Log_2_ H3K27ac signal at ATAC peaks in C57 BMDMs under basal and IL-4 conditions. FC, fold change. (**B**) Comparison of H3K27ac signal between C57 and BALB or SPRET under the 24-hour IL-4 condition. (**C**) Log_2_ H3K27ac fold changes after 24-hour IL-4 in C57 versus other strain in enhancers. (**D**) Distributions of IL-4 H3K27ac log_2_ fold changes. Levene’s test was performed to test response differences in conventional versus super enhancers. (**E**) *Ak2* super enhancer responsive to IL-4 and conserved across all strains. (**F**) *Msx3* IL-4–induced enhancer in C57, BALB, and NOD, but not PWK and SPRET BMDMs. Absolute DeepLIFT scores indicate predicted importance of single nucleotides for enhancer activity. Dashed lines represent locations of PWK or SPRET variants. (**G** and **H**) Enhancers were categorized into strain-similar and strain-differential on the basis of fold differences in H3K27ac between C57 and one of the other strains. Table with percentages of enhancers containing local genetic variants (G) and the percentage of enhancers that contain predicted functional variants (H). (**I**) Log_2_-scaled enrichment of enhancers with variants at top-scoring positions based on DeepLIFT scores. The enrichment was calculated by (% enhancers in one category with top variants) / (% all enhancers with top variants). (G) and (H) are based on the top 100 and 20%, respectively.

To investigate the impact of cis variation on putative transcriptional regulatory elements, we defined high-confidence IL-4–activated enhancers as intronic or intergenic open chromatin regions [based on assay for transposase-accessible chromatin using sequencing (ATAC-seq)] with at least 2.5-fold increase in H3K27ac (*23*) and RNA Pol II (*24*) after IL-4 treatment (fig. S2, A to D). In 24-hour IL-4–stimulated C57 BMDMs, 1093 regions exhibited a >2.5-fold increase in H3K27ac, whereas 441 regions exhibited a >2.5-fold decrease, corresponding to putative IL-4–activated and IL-4–repressed enhancers, respectively (Fig. 2A). Comparison of C57 enhancers to those of other strains under IL-4 treatment conditions revealed marked differences that scaled with the degree of genetic variation (Fig. 2B and fig. S2, E and F). We further subdivided these regions into “conventional enhancers” (Fig. 2C, blue) and “super enhancers” (Fig. 2C, orange), on the basis of the density distribution of normalized H3K27ac tag counts (*25*). Super enhancers represent regions of the genome that are highly enriched for cell-specific combinations of transcription factors and coregulators and control the expression of genes required for cellular identity and critical functions. In comparison to conventional enhancers, super enhancers exhibited significantly less variation in H3K27ac in response to IL-4 (Fig. 2D and fig. S2G). For example, IL-4 induction of the *Ak2* super enhancer (Fig. 2E) is highly conserved between the five strains. In contrast, a typical example of strain specificity is provided by the conventional enhancers associated with the *Msx3* gene. These enhancers are IL-4 inducible only in BALB, C57, and NOD and absent in PWK and SPRET macrophages (Fig. 2F).

We next compared the fractions of enhancers containing variants in strain-similar enhancers (<1.5-fold differences in H3K27ac between strains) to strain-differential enhancers at increasing levels of difference (fold differences >1.5 to >4; Fig. 2G). The fraction of enhancers containing variants at strain-similar enhancers ranged from 17 to 19% in the strains most similar to C57 (BALB and NOD) to 69 to 93% in the most genetically divergent strains (PWK and SPRET). As expected, the fraction of enhancers containing variants increased with increasing levels of difference, except for SPRET that may have reached a saturation of variation capacity (Fig. 2G). These findings are not only consistent with local variants affecting enhancer activity but also indicate that a substantial fraction of even strongly strain-differential IL-4–induced enhancers lack these variants, consistent with previous findings for strain-specific enhancers overall (*14*).

In an effort to distinguish silent variants from those affecting enhancer activities, we trained a DeepSEA convolutional neural network to classify enhancers as active or inactive under the 24-hour IL-4 condition on the basis of local sequence context (*26*). The training data consisted of enhancers active under IL-4 conditions (positive data) and random background (negative data). The area under the receiver operating characteristic curve (auROC) was 0.894 on test data. We then used DeepLIFT (*27*) to compute the importance score of each nucleotide on the basis of the model’s classification decision. Variants at positions with top importance scores within surrounding 300–base pair (bp) enhancer regions are hypothesized to affect enhancer activity. We considered variants residing in the top 20% of importance scores for each region as predicted functional variants. The *Msx3* enhancer in Fig. 2F illustrates 4 predicted functional variants of 14 variants in PWK and SPRET (red dashed lines). By focusing on top-scoring variants rather than all local variants, we saw an expected overall decreased percentage of enhancers with top-scoring variants (Fig. 2H and fig. S2H). On the other hand, enrichment of predicted functional variants increases as a function of importance score threshold and is strongest for enhancers that show the highest differences across strains (Fig. 2I). This is true when considering all strains, including SPRET. These results reveal a quantitative impact of variants affecting enhancer under IL-4 treatment conditions and suggest the extent to which a deep learning approach can distinguish potentially functional variants from the silent variants.

### IL-4–activated enhancers use preexistent promoter-enhancer interactions to regulate gene activity

**Fig. 3:**
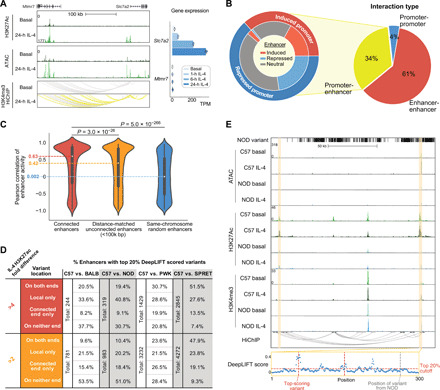
IL-4 enhancers use preexistent promoter-enhancer interactions to regulate gene activity. (**A**) HiChIP indicates that the *Slc7a2* promoter is highly connected with several IL-4–activated enhancers. *Slc7a2* and *Mtmr7* gene expression upon IL-4 stimulation was shown. (**B**) Different categories of HiChIP interactions (right) and enhancer-promoter connections overlapping with IL-4–responsive regulatory elements in C57 BMDMs (left). Outer ring indicates induced or repressed promoters, while inner ring indicates their connected enhancers associated with IL-4–induced, IL-4–repressed, or IL-4–neutral H3K27ac. (**C**) Correlations of H3K27ac signal between connected enhancers compared to noninteractive enhancers using Mann-Whitney *U* test. (**D**) Table representing enhancers containing DeepLIFT high-scored genetic variants locally or at connected elements in pairwise comparisons between C57 and other strains. (**E**) Strain-differential enhancer between C57 and NOD where genetic variants were absent locally but present at a connected enhancer with two DeepLIFT high-scored variants (red dashed lines).

Interpretation of effects of genetic variation on distal regulatory elements is facilitated by knowledge of cell-specific enhancer-promoter interactions (*28*). To identify connections of IL-4–responsive enhancers to target promoters, we performed HiChIP using an antibody to H3K4me3 (*29*) in C57 BMDMs under basal conditions and after 24 hours of IL-4 treatment. HiChIP interactions are exemplified in Fig. 3A at the *Slc7a2* locus, a gene that becomes maximally activated after 24 hours of IL-4 treatment and connects primarily to an enhancer-like region within the *Mtmr7* gene, which itself is expressed at negligible levels (Fig. 3A). Although we observed instances of IL-4–specific interactions (e.g., yellow loops), a differential interaction analysis was unable to identify significantly different interactions between basal and IL-4 conditions, supported by the high correlation of interaction intensity between the two conditions (fig. S3A). Moreover, enhancer-promoter interaction intensity did not correlate with IL-4–induced gene activity or the level of H3K4me3 at promoters (fig. S3, B and C). However, IL-4–activated promoters mostly interact with IL-4–activated enhancers (Fisher’s exact test, *P* = 2.2 × 10^−16^), and IL-4–repressed promoters strongly interact with IL-4–repressed enhancers (*P* = 1.2 × 10^−15^; Fig. 3B). These results suggest a preexistent and relatively stable landscape of enhancer-promoter interactions in macrophages, whose regulatory function was activated in response to IL-4.

Although the HiChIP assay is designed to capture promoter-enhancer interactions based on preferential occurrence of H3K4me3 at promoters, we also recovered 145,907 pairs of interactive enhancers (Fig. 3B), consistent with more than one enhancer being in local proximity of a target promoter. The H3K27ac correlations between interactive enhancers were significantly stronger than those between noninteractive enhancers (Fig. 3C and fig. S3D), consistent with their being functionally related. Noticeably, the closer enhancers, despite being noninteractive on the basis of our data, still have much stronger correlation than completely random enhancers, which might be due to more frequent contacts of nearby regions within the same interactive domain that were not captured by H3K4me3 HiChIP. On the basis of the high correlation of enhancer activity between connected enhancers, we hypothesized that enhancer-enhancer interactions could explain strain-differential enhancer when local genetic variants were absent (Fig. 2H). Among 224 interactive enhancers exhibiting a >4-fold difference in H3K27ac signal between BALB and C57 under the IL-4 condition, the original ~50% of strain-differential enhancers with predicted functional variants was further split into 20.5% that had top-scoring variants on both ends and 33.6% that had only local top-scoring variants (Fig. 3D, upper left). Depending on the strain comparison, an additional 8.2 to 19.9% of differential enhancers could be explained by genetic variants in interacting enhancers, indicating that enhancers may be affected by functional variants in other connected enhancers. Reducing the fold change requirement to twofold yielded a smaller proportion of strain-differential enhancers containing local variants overall but significantly increased the proportion having top-scoring variants on the connected ends only (15.4 to 26.5%, Fisher’s exact test *P* = 0.002 for BALB, 2.8 × 10^−5^ for NOD, 8.3 × 10^−7^ for PWK, and 3.3 × 10^−10^ for SPRET), suggesting that local variants have a stronger effect on inducing differential activation than variants at connected enhancers (Fig. 3D, bottom, and fig. S3E). Figure 3E illustrates an enhancer affected by genetic variants at the connected enhancer. The enhancer highlighted on the left is notably more active in C57 than NOD. This region lacks local variants in NOD but is connected to another enhancer ~100 kb away (highlighted on the right) containing multiple variants that are predicted to affect activity by deep learning. These findings are consistent with genetic variants at an enhancer influencing the activity states of other enhancers that lack local functional variants within the same connected network (*30*, *31*).

### Motif mutation analysis identifies motifs that are functionally associated with IL-4–induced enhancer activity

**Fig. 4:**
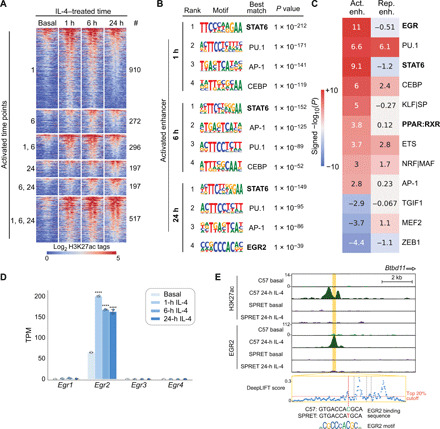
Motif analysis identifies motifs functionally associated with IL-4–induced enhancers. (**A**) Heatmap showing the effects of 1-, 6-, and 24-hour IL-4 stimulation on enhancer activation based on H3K27ac abundance. (**B**) Top motifs enriched at ATAC-seq peaks exhibiting gained H3K27ac at different time points. (**C**) MAGGIE motif mutation analysis on strain-differential activated and repressed enhancers after 24-hour IL-4. (**D**) *Egr* gene expression in C57 BMDMs under basal conditions and after stimulation with IL-4, ****q < 0.0001, compared to basal. (**E**) Example of a strain-differential activated enhancer upstream of the *Btbd11* gene based on IL-4–induced H3K27ac signal in C57 but not in SPRET BMDMs, supported by binding of EGR2 and a functional variant predicted by DeepLIFT that mutates the EGR2 motif.

IL-4 rapidly activates a set of enhancers, most of which exhibit maximal H3K27ac at 1 or 6 hours and returns to (near) basal levels by 24 hours (Fig. 4A, top three clusters) when most gene expression changes were found (fig. S1B). Others are long-lasting or become activated at later time points (Fig. 4A, bottom three clusters). De novo motif enrichment analysis of enhancers exhibiting >2.5-fold increase in H3K27ac and RNA Pol II at 1, 6, and 24 hours (fig. S2A) recovered a STAT6 motif as the most enriched motif for all time points (Fig. 4B). Motifs for the lineage-determining factors PU.1 and AP-1 (activator protein 1) family members were also recovered in all three classes of enhancers. Notably, an EGR2 motif was significantly enriched among enhancers induced at 24 hours.

As a genetic approach to identify functional transcriptional factor binding motifs, we assessed the quantitative impact of the genetic variation provided by the five different strains of mice on the IL-4 response of enhancers using the motif mutation analysis tool MAGGIE. MAGGIE associates changes of epigenomic features at homologous sequences (e.g., enhancer activation or enhancer repression) with motif mutations caused by genetic variation so that it can prioritize motifs that likely contribute to the regulatory function (*32*). This analysis identified more than a dozen motif clusters in which motif mutations were significantly associated with strain-differential IL-4–activated or IL-4–repressed enhancers (Fig. 4C and fig. S4A). The EGR motif was found as the top motif associated with enhancer activation at the 24-hour treatment time, as well as motifs of known SDTFs STAT6 and PPARγ and macrophage LDTFs PU.1, AP-1, and C/EBP (CCAAT/enhancer binding protein) (Fig. 4C). We also found Kruppel-like factor (KLF) motifs associated with IL-4 enhancer activation, which fits with increased KLF4 expression by IL-4 (fig. S4B), and an nuclear factor erythroid 2-related factor (NRF) motif associated with both enhancer activation (Fig. 4C).

The identification of STAT6 and PPARγ motif mutations as being functionally associated with strain-differential IL-4 activation is consistent with substantial prior work demonstrating the importance of these factors in regulating IL-4–dependent gene expression (*21*, *33*). Out of the EGR family members, only *Egr2* is expressed in unstimulated BMDMs and rapidly induced after IL-4 stimulation (Fig. 4D and fig. S4C). *Egr2* has also been associated with late IL-4 enhancer activation in a recent study (*22*). Examination of the *Egr2* locus indicates IL-4–induced binding of STAT6 and PPARγ to a set of upstream super enhancers that gain H3K27ac and RNA Pol II signal after IL-4 stimulation (fig. S4D). These super enhancers were observed in BMDMs of all five different strains (fig. S4E) that are strongly connected to the *Egr2* promoter in C57 BMDMs as indicated by H3K4me3 HiChIP interactions. Overall, these findings suggest a functionally important role of EGR2 in contributing to IL-4–induced enhancer activation in BMDMs.

### IL-4–induced EGR2 contributes to late IL-4 enhancer activation

To incorporate EGR2 into a comprehensive analysis of the impact of genetic variation on IL-4 responses, we next performed ChIP-seq for EGR2 under basal and 24-hour IL-4 treatment conditions. This confirmed the prediction that mutations in EGR binding sites contribute to strain-differential enhancer activation by altering the binding of EGR2. An example is provided by the *Btbd11* enhancer, which is IL-4 inducible in C57, but not in SPRET BMDMs (Fig. 4E). Consistent with the loss of EGR2 binding in SPRET, a C-to-T variant in SPRET mutated an EGR2 motif.

**Fig. 5:**
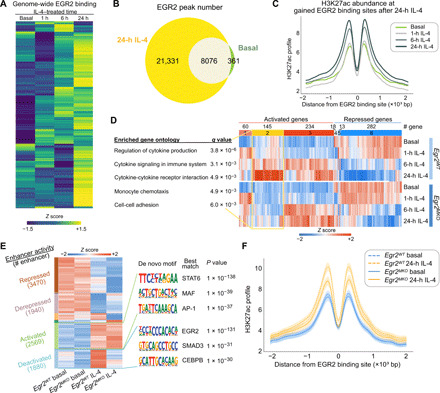
IL-4–induced EGR2 contributes to late IL-4 enhancer activation. (**A**) Heatmap displaying EGR2 ChIP-seq binding intensity after IL-4 stimulation over time in C57 BMDMs. (**B**) Number of EGR2 binding sites after 24-hour IL-4 compared to the basal condition in C57 BMDMs. (**C**) H3K27ac profiles at 24-hour IL-4–induced intergenic and intronic EGR2 peaks in C57 BMDMs. (**D**) Expression of IL-4–regulated genes in *Egr2^WT^* and *Egr2^MKO^* BMDMs with top gene ontology terms displayed for cluster 2. (**E**) Enhancer activity of IL-4–regulated enhancers in *Egr2^WT^* and *Egr2^MKO^* BMDMs. Enriched motifs at EGR2-dependent and EGR2-independent IL-4–induced enhancers using each other as backgrounds. (**F**) H3K27ac profiles at IL-4–induced EGR2 binding sites in *Egr2^WT^* and *Egr2^MKO^* BMDMs. Ninety percent confidence intervals are shown together with the average profiles.

To study EGR2 binding and its effects on gene regulation over time, we additionally measured EGR2 binding at 1 and 6 hours after IL-4. We saw a marked expansion of the EGR2 cistrome after stimulation with IL-4 (Fig. 5, A and B). Most EGR2 binding sites had an increasing binding intensity over time and reached maximum values at 24 hours (Fig. 5A and fig. S5A). In contrast, STAT6 binding was strong immediately after 1 hour and slowly decreased in its binding intensity (fig. S5A). At 24 hours, there were over 20,000 newly gained EGR2 binding sites (Fig. 5B), which were associated with an increase in H3K27ac and RNA Pol II signals over time, supporting a major role of EGR2 in late enhancer activation (Fig. 5C and fig. S5B).

To extend these analyses, we crossed *Egr2^flfl^* (*34*) (*Egr2^WT^*) with *LyzM*-Cre^+^ mice to obtain *LyzM*-Cre^+^ *Egr2^flfl^* (*Egr2^MKO^*) mice. This resulted in efficient deletion of *Egr2* in BMDMs (fig. S5, C and D). RNA-seq data from *Egr2^WT^* and *Egr2^MKO^* BMDMs indicated that at the mRNA level, EGR2 is regulating ~40% of the 24-hour IL-4–induced genes (Fig. 5D, fig. S5E, and table S3), many of which correspond to gene ontology terms related to cytokine production and signaling (Fig. 5D). At the regulatory level, we found that ~40% of the IL-4–induced enhancers at 24 hours had notably decreased activity in *Egr2^MKO^* BMDMs (Fig. 5E, blue cluster, and fig. S5, F to H). In concordance, the IL-4 induction in H3K27ac was found to be decreased at IL-4–induced EGR2 binding sites in *Egr2^MKO^* BMDMs (Fig. 5F). On the basis of a motif enrichment analysis of the EGR2-dependent IL-4–activated enhancers (blue cluster “deactivated”) with the EGR2-independent enhancers as background (green cluster “activated”), we found EGR2 as the most significantly enriched motif and SMAD3 and C/EBP motifs as the second and the third hits (Fig. 5E). When comparing the EGR2-independent activated enhancers (activated) to the EGR2-dependent ones (deactivated), the STAT6 motif was most significantly enriched (Fig. 5E), suggesting that STAT6 could work independently of EGR2 to maintain the late activation for a subset of enhancers. Together, these findings establish an essential role of EGR2 in IL-4–dependent enhancer activation and gene expression and are in full agreement with the recent studies of Daniel *et al.* (*22*).

### Collaborative and hierarchical transcription factors interact at IL-4–dependent enhancers

**Fig. 6:**
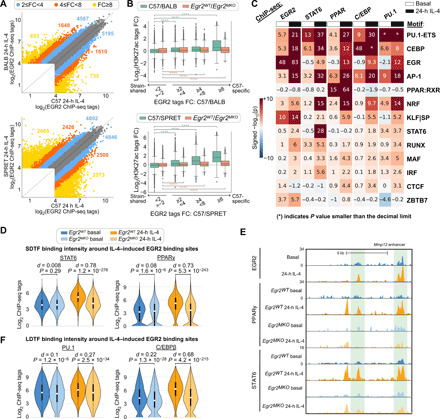
Collaborative and hierarchical transcription factors interact at IL-4 enhancers. (**A**) Scatter plots comparing binding of EGR2 in C57 versus BALB and C57 versus SPRET IL-4–stimulated BMDMs. (**B**) Log_2_ fold changes of H3K27ac signal between different strains (green boxes) or between *Egr2^WT^* and *Egr2^MKO^* BMDMs (orange boxes) at C57-specific and strain-shared EGR2 binding sites. Distributions of C57-specific sites were compared to those of strain-shared sites in the same category using the two-sample *t* test. ****P < 0.0001. (**C**) Functional motifs from MAGGIE analysis at EGR2, STAT6, PPARγ, C/EBPβ, and PU.1 binding sites. (**D**) STAT6 and PPARγ binding at IL-4–induced EGR2 peaks in *Egr2^WT^* and *Egr2^MKO^* BMDMs. Cohen’s *d* effect size and *P* values from Mann-Whitney *U* tests are shown. (**E**) Cobinding of STAT6, EGR2, and PPARγ at the *Mmp12* enhancer. (**F**) C/EBPβ and PU.1 binding at IL-4–induced EGR2 peaks in *Egr2^WT^* and *Egr2^MKO^* BMDMs.

Analysis of the genome-wide binding patterns of EGR2, STAT6, and PPARγ indicated intensive cobinding at IL-4–activated enhancers (fig. S6A). To achieve highly strain-differential responses to IL-4 that are observed at the level of gene expression (Fig. 1) and enhancer activation (Fig. 2), we hypothesize these factors to exert their transcriptional effects via correspondingly divergent genomic binding patterns. About 4000 EGR2 binding sites exhibited more than fourfold differences in normalized tag counts between C57 and BALB and about 10,000 between C57 and SPRET (Fig. 6A). Similar relationships are observed for STAT6 (fig. S6B). Within the strain comparisons of C57 to BALB and SPRET, C57-specific EGR2 binding sites are associated with stronger H3K27ac signal in C57 (Fig. 6B, green boxes). At these C57-specific EGR2 binding sites, H3K27ac signal is more strongly down-regulated in *Egr2^MKO^* macrophages (Fig. 6B, orange boxes). An example of this is demonstrated in fig. S6C in which C57-specific binding of EGR2 is associated with an IL-4–induced increase in H3K27ac, which is absent in SPRET (where the EGR2 motif is disrupted; Fig. 4E) and is decreased in *Egr2^MKO^* BMDMs.

The strain-differential binding patterns of SDTFs and LDTFs enabled motif mutation analysis to study the importance of motifs for SDTF and LDTF binding (Fig. 6C). As a validation, LDTF and SDTF binding depended on their own motifs (e.g., PU.1 motif mutation was significantly associated with PU.1 binding, indicating that when PU.1 binding is lost in one strain, it is often found that the PU.1 motif score is reduced in that strain compared to the other). In addition, mutations in the motifs of LDTFs PU.1, C/EBP, and AP-1 influence the binding of all LDTFs and SDTFs, which fits with earlier observations (*13*). We found that the PPAR motif is only significant for PPARγ binding, and likewise, the STAT6 motif is not associated with binding of other SDTFs or LDTFs but STAT6. We found that mutations in EGR2 motifs are significantly associated with binding of SDTFs STAT6 and PPARγ and LDTFs PU.1 and C/EBPβ under IL-4 conditions, but not under basal conditions. These analyses also provided evidence for functional roles of several additional transcription factors. Mutations in NRF motifs were strongly associated with the IL-4–dependent binding of all SDTFs and LDTFs. Both NRF1 and NRF2 are expressed in BMDMs (fig. S4B) and are involved in lipid metabolism and stress responses (*35*–*37*). Mutations in KLF motifs were strongly associated with EGR2 binding under both basal and IL-4 conditions. KLF2, KLF4, and KLF6 are expressed in BMDMs (fig. S4B), and KLF4 has previously been associated with anti-inflammatory roles in macrophages (*38*). Mutations in IRF motifs were moderately associated with IL-4–dependent STAT6 binding. Multiple IRFs, including IRF4, are expressed in BMDMs (fig. S6D), and IRF4 has previously been linked to macrophage polarization by IL-4 (*39*, *40*).

A prediction emerging from the analysis results above is that EGR2 should have a small effect on the cobinding of SDTFs and LDTFs under basal conditions and a substantial effect following 24 hours of IL-4 treatment. To examine this prediction, we performed ChIP-seq for STAT6, PPARγ, PU.1, and C/EBPβ in *Egr2^WT^* and *Egr2^MKO^* BMDMs and evaluated their binding in the vicinity of IL-4–induced EGR2 binding sites. Deletion of *Egr2* had little effect on PPARγ and STAT6 binding under basal conditions and a much greater effect following 24 hours of IL-4 treatment (Fig. 6D). As an example, in *Egr2^MKO^* BMDMs, PPARγ and STAT6 binding was found decreased at the *Mmp12* enhancer at sites where EGR2 normally binds (Fig. 6E). Similarly, PU.1 and C/EBPβ binding was more significantly affected by *Egr2* deletion under the IL-4 condition than the basal condition (Fig. 6F). In concert, these findings provide evidence for collaborative interactions between PU.1, C/EBPs, AP-1, STAT6, PPARγ, and EGR2 as major drivers of late enhancer activation in response to IL-4. EGR2 is a strong collaborative factor as it promotes binding of LDTFs PU.1 and C/EBPβ and SDTFs STAT6 and PPARγ after IL-4 stimulation.

### Quantitative variations in motif affinity determine dynamic responses of IL-4 enhancers

**Fig. 7:**
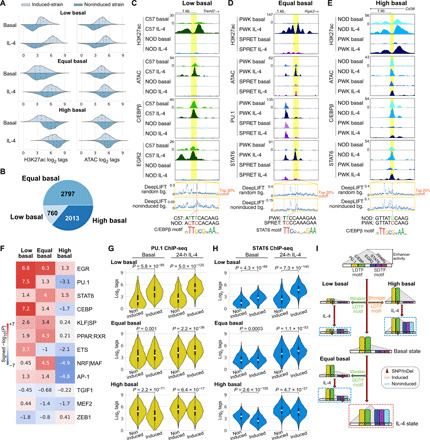
Quantitative variations in motif affinity determine dynamic responses of IL-4 enhancers. (**A**) Three different categories of strain-differential IL-4–activated enhancers with distributions of ATAC and H3K27ac signal. Dashed lines in each distribution indicate quartiles. (**B**) Numbers of enhancers in the three categories. (**C** to **E**) Example of low (C), equal (D), and high (E) basal enhancers with high-impact variants predicted by DeepLIFT. (**F**) MAGGIE motif mutation analysis on different categories of enhancers. (**G** and **H**) Binding intensities of PU.1 (G) and STAT6 (H) in noninduced and induced strains at different categories of enhancers. (**I**) Graphical representation of the general mechanisms for different categories of IL-4–induced enhancers. SNP, single-nucleotide polymorphism; InDel, insertion/deletion.

We next investigated the possibility that the mutational status of the dominant motifs recovered by MAGGIE analysis was sufficient to predict qualitative patterns of strain-differential responses of IL-4–induced enhancers. Following the classification of strain-differential mRNA responses (Fig. 1), we used H3K27ac to define three different categories of strain-differential IL-4–induced enhancers (Fig. 7A, left column): enhancers exhibiting lower levels of basal activity in the lowly induced strain (low basal); enhancers with a similar level of basal activity (equal basal); and enhancers in which a lack of IL-4–induced activity was associated with relatively higher basal activity compared with the more responsive strain (high basal). Using these criteria, we identified 760 low basal, 2797 equal basal, and 2013 high basal enhancers from all pairwise comparisons of the five strains that exhibited >2-fold differences in H3K27ac induction (Fig. 7B). The closest genes for enhancers of these three categories follow similar trends as observed for enhancer activity (fig. S7A). Low basal, equal basal, and high basal enhancers are exemplified by enhancers associated with the *Treml2*, *Ripk2*, and *Cd36* genes, respectively (Fig. 7, C to E, and fig. S7, B to D).

Consideration of chromatin accessibility as determined by ATAC-seq further uncovered potential mechanisms that distinguished the three enhancer categories (Fig. 7A, right column). The enhancers in the low basal category showed low to absent basal ATAC signal in noninduced strains, suggesting a lack of LDTFs under the basal condition to preoccupy chromatin required for subsequent recruitment of SDTFs after IL-4 stimulation. In contrast, high basal enhancers exhibited a higher basal level of ATAC in noninduced strains compared with the induced strains (Fig. 7A, right column), suggesting stronger LDTF binding in noninduced strains under the basal condition. Different from the other categories, equal basal enhancers exhibited similar levels of chromatin accessibility under both basal and IL-4 conditions between comparative strains, suggesting that the recruitment of SDTFs might be the key determinant for the strain difference instead of basal LDTF binding.

To test the hypotheses above regarding the different determinants for the three categories of enhancers, we performed MAGGIE motif mutation analysis on each category of enhancers that contain motif mutations (fig. S7E). We found that mutations in motifs of LDTFs PU.1/ETS and C/EBP were associated with low basal enhancers and resulted in better motifs in induced strains, while mutations in motifs of SDTFs EGR, STAT6, PPAR, and NRF/MAF were associated with the equal basal category leading to better motifs in induced strains (Fig. 7F and fig. S7F). Mutations in EGR motifs were also associated with the low basal category, suggesting another role of EGR2 as a strong collaborative factor under the IL-4 condition, which is supported by the notable decrease in open chromatin under IL-4 conditions after deletion of *Egr2* (fig. S5F). Of particular interest, the high basal category of enhancers was most strongly associated with negative significance scores for LDTF PU.1, C/EBP, and AP-1 as well as NRF/MAF, meaning higher motif affinity in noninduced strains (Fig. 7F).

We validated these findings with our ChIP-seq data by examining the binding profiles of PU.1, C/EBPβ, STAT6, PPARγ, and EGR2 in three categories of enhancers. In low basal enhancers, we saw significantly reduced binding of PU.1 and C/EBPβ in noninducible strains under both basal and IL-4 conditions (Fig. 7G and fig. S7G). This pattern was accompanied by significantly weaker binding of SDTFs STAT6, EGR2, and PPARγ after IL-4 stimulation (Fig. 7H and fig. S7H). The example in Fig. 7C showed the absence of C/EBPβ binding in NOD under the basal condition likely due to two local variants at high-scored positions according to DeepLIFT that together mutated a C/EBP motif. Upon IL-4 stimulation, neither C/EBPβ nor EGR2 was further recruited. For equal basal enhancers, we found that PU.1 and C/EBPβ binding was similar under basal conditions in induced and noninduced strains (Fig. 7G and fig. S7G). Upon IL-4 stimulation, the induced strains displayed significantly stronger binding of SDTFs STAT6, EGR2, and PPARγ (Fig. 7H and fig. S7H). In the example in Fig. 7D, STAT6 binding was strongly induced by IL-4 at the *Ripk2* enhancer in PWK but was absent in SPRET. Despite the clear difference in STAT6 binding, none of the local variants between the two strains were predicted functional when using a neural network model trained with random genomic backgrounds. To better capture the sequence patterns relevant for enhancer activation, we retrained neural networks using noninduced enhancers as the background, which emphasized a relatively divergent set of *k*-mers and focused less on those matched with LDTF motifs (fig. S7I). As a result, our retrained model assigned a high DeepLIFT score to one of the nucleotides in a STAT6 motif that was mutated by a variant in SPRET (Fig. 7D). For high basal enhancers, we found stronger binding of not only the LDTFs PU.1 and C/EBPβ (Fig. 7G and fig. S7G) but also the SDTFs STAT6 and PPARγ (Fig. 7H and fig. S7H) in noninduced strains under basal conditions. For example, high basal levels of C/EBPβ and STAT6 binding were observed at the *Cd36* enhancer in NOD mice (Fig. 7E). The only local variant in PWK was at a predicted functional position and mutated a C/EBP motif likely causing the low basal C/EBPβ binding in PWK. In concert, these analyses validated the importance of LDTF motif mutations as primary determinants of differential enhancer activation in low basal and high basal enhancers, while also demonstrating the expected consequences of SDTF motif mutations in determining strain-differential activation of equal basal enhancers (Fig. 7I).

## DISCUSSION

Here, we report a systematic investigation of the effects of natural genetic variation on signal-dependent gene expression by exploiting the highly divergent responses of BMDMs from diverse strains of mice to IL-4. Unexpectedly, despite broad conservation of IL-4 signaling pathways and downstream transcription factors in all five strains, only 26 of more than 600 genes observed to be induced >2-fold by IL-4 at 24 hours reached that level of activation in all five strains, and more than half were induced in only a single strain. To the extent that this remarkable degree of variation observed in BMDMs occurs in tissue macrophages and other cell types in vivo, it is likely to have substantial phenotypic consequences with respect to innate and adaptive immunity, tissue homeostasis, and wound repair. Notably, only ~25% of the variation in response to IL-4 was due to altered dynamic ranges in the context of an equivalent level of basal expression. Nearly half of the genes showing strain-specific impairment in IL-4 responsiveness exhibited low basal activity, whereas lack of induction was associated with constitutively high basal levels of expression in the remaining ~25%. These qualitatively different patterns of strain responses to IL-4 imply distinct molecular mechanisms by which genetic variation exerts these effects.

Motif mutation analysis of strain-differential enhancer activation recovered a dominant set of motifs recognized by known LDTFs PU.1, C/EBPβ, and AP-1 family members, as well as motifs recognized by SDTFs STAT6 and PPARγ that have been previously established to play essential roles in the IL-4 response. In addition, effects of mutations in motifs for EGR, NRF, and KLF also strongly implicate these factors as playing important roles in establishing basal and induced activities of IL-4–responsive enhancers, which was genetically confirmed for EGR2 in this study and a recent study by Daniel *et al.* (*22*). It will be of interest in the future to perform analogous studies of NRF and KLF factors.

Analysis of strain-differentially activated enhancers revealed qualitative differences in basal and IL-4–dependent activity that were analogous to the qualitative differences observed for strain-differentially activated genes. As expected, sequence variants reducing the affinity of SDTFs STAT6, PPARγ, and EGR2 were the major forms of variation resulting in strain-differential IL-4 induction of equal basal enhancers. From the standpoint of interpreting the effects of noncoding variation, these types of sequence variants are silent in the absence of IL-4 stimulation. As also expected, sequence variants strongly reducing the binding affinity of LDTFs prevented the generation of open chromatin required for subsequent binding of SDTFs. These variants are thus expected to result in loss of enhancer function in a signal-independent manner. Of particular significance, these analyses also provide strong evidence that quantitative variation in suboptimal motif scores for LDTFs is a major determinant of differences in the absolute levels and dynamic range of high basal enhancers across strains. The importance of low-affinity motifs in establishing appropriate quantitative levels of gene expression within a given cell type and cell specificity across tissues has been extensively evaluated (*41*–*43*). Here, we present evidence that improvement of low-affinity motifs for LDTFs not only increases basal binding of the corresponding transcription factor but also is associated with increased basal binding of STAT6 and PPARγ, thereby rendering their actions partially or fully IL-4 independent. These findings thus provide evidence that quantitative effects of genetic variation on LDTF motif scores play major roles in establishing different absolute enhancer activity levels and dynamic ranges of their responses to IL-4 that are observed between strains.

To go beyond the discovery of mechanisms mediating the IL-4 response using natural genetic variation, a major objective of these studies was to use the resulting datasets as the basis for interpreting and predicting the effects of specific variants. As expected, enhancers exhibiting strain-specific differences in IL-4 responses were significantly enriched for sequence variants. However, the background frequencies of variants in the much larger sets of strain-similar enhancers ranged from 17 to 93%, consistent with the vast majority of these variants being silent and underscoring the challenges of discriminating them from functional variants. The application of recently developed deep learning approaches illustrates both the potential of these methods to improve predictive power and their current limitations. Nucleotides predicted by DeepLIFT to be of functional importance frequently intersected with variants at strain-differential enhancers that significantly altered LDTF or SDTF motifs, with over eightfold enrichment in enhancers with strongest strain differences (top 1% variants for C57 versus BALB comparison; Fig. 2I), strongly suggesting causality. Although DeepLIFT scored a substantial fraction of variants present in strain-similar enhancers with low importance, a large fraction of remaining strain-similar enhancers contained variants associated with high DeepLIFT scores, most likely representing false positives. Furthermore, we found that the highest scoring variants in some cases depended on the choice of data used to train the convolutional neural network (e.g., using random versus noninduced enhancers as negative training examples). This observation has important implications with respect to application of deep learning models to identify potential functional variants in disease contexts. The datasets generated by these studies will therefore provide an important resource for further improvements in methods for interpretation of local genetic variation.

These analyses further indicated that 20 to 50% of the most divergent IL-4–responsive enhancers lacked any functional variants in the proximity of open chromatin. This fits with previous observations that variant-free enhancers can reside in cis-regulatory domains (CRD) containing functionally interacting enhancers, suggesting that a variant strongly affecting one enhancer within the CRD could have domain-wide effects (*14*). This concept was supported and extended here by HiChIP experiments. In addition to demonstrating that the IL-4 response was primarily associated with preexisting enhancer-promoter connections, the HiChIP assay also captured a large number of enhancer-enhancer interactions. Examination of these connected enhancers provided evidence that a substantial fraction of strain-differential enhancers lacking local variants were connected to strain-differential enhancers containing functional variants. An important future direction will be to further investigate the significance and mechanisms underlying these associations.

Collectively, these studies reveal general mechanisms by which noncoding genetic variation influences signal-dependent enhancer activity, thereby contributing to strain-differential patterns of gene expression and phenotypic diversity. A major future goal will be to incorporate these findings into improved algorithms for prediction of absolute levels and dynamic responses of genes to IL-4 at the level of individual genes.

## MATERIALS AND METHODS

### Experimental design

To investigate the influence of genetic variation on signal-dependent gene expression, enhancer activation, and transcription factor binding, we performed RNA-seq, ATAC, and ChIP-seq to study the responses of macrophages derived from five different inbred mouse (C57, BALB, NOD, PWK, and SPRET) strains to the anti-inflammatory cytokine IL-4 (Fig. 1A).

### Mice

Female and male breeder mice for C57, BALB, NOD, PWK, and SPRET mice were purchased from the Jackson laboratory. F1 C57 × SPRET mice were crossed, and *Egr2^fl/fl^* mice were generously donated by Drs. V. Lazarevic and L. Warren (National Institutes of Health) and crossed to *LyzM*-Cre mice (the Jackson laboratory) to achieve myeloid-specific targeted deletion of *Egr2*. Mice were housed at the University of California San Diego animal facility on a 12-hour/12-hour light/dark cycle with free access to normal chow food and water. All animal procedures were in accordance with University of California San Diego research guidelines for the care and use of laboratory animals. Eight- to 12-week-old healthy female mice were used for all our experiments.

### BMDM culture

Femur, tibia, and iliac bones from the different mouse strains were flushed with Dulbecco’s modified Eagle’s medium (DMEM) high glucose (Corning), and red blood cells were lysed using red blood cell lysis buffer (eBioscience). After counting, 20 million bone marrow cells were seeded per 15-cm nontissue culture plates in DMEM high glucose (50%) with 20% fetal bovine serum (FBS; Omega Biosciences), 30% L929 cell–conditioned laboratory-made media [as source of macrophage colony-stimulating factor (M-CSF), as described before (*14*)], penicillin/streptomycin + l-glutamine (100 U/ml; Gibco), and amphotericin B (2.5 μg/ml; HyClone). After 4 days of differentiation, mouse M-CSF (16.7 ng/ml; Shenandoah Biotechnology) was added to the media. After an additional 2 days of culture, adherent cells were scraped and subsequently seeded onto tissue culture–treated petri dishes in DMEM containing 10% FBS, penicillin/streptomycin + l-glutamine (100 U/ml), amphotericin B (2.5 μg/ml), and M-CSF (16.7 ng/ml). Macrophages were left untreated or treated with mouse recombinant IL-4 (20 ng/ml; PeproTech) for 1, 6, or 24 hours.

### Immunofluorescence

Cells were fixed with Cytofix/Cytoperm Buffer (BD Biosciences, BD554714) for 10 min at room temperature. Cytofix/Cytoperm buffer was removed, and cells were washed twice with Hanks’ balanced salt solution containing 2% bovine serum albumin (BSA) and 1 mM EDTA. Cells were kept in permeabilization/wash buffer (BD Biosciences, BD554714) for 1 hour at 4°C or until the experiment was performed. Fixed cells were blocked using 3% BSA, 0.1% Triton–phosphate-buffered saline (PBS) for 30 min at room temperature and then with 1/200 of the EGR2 antibody (Abcam) overnight at 4°C. The next day, cells were washed with 0.1% Triton-PBS and incubated with 1/200 donkey anti-rabbit 555 (Thermo Fisher Scientific, no. A31572) secondary antibody and phalloidin (Abcam, ab176759) for staining actin filaments, and nuclei were counterstained with 4′,6-diamidino-2-phenylindole. After washing with 0.1% Triton-PBS, slides were mounted with ProLong Gold Antifade Reagent (Life Technologies, no. 10144). Images were taken using a Leica SP8 with light deconvolution microscope.

### RNA-seq library preparation

Total RNA was isolated from cells and purified using RNA Direct-zol microprep columns and ribonuclease (RNase)–free deoxyribonuclease digestion according to the manufacturer’s instructions (Zymo Research). Sequencing libraries were prepared in biological replicates from polyadenylate [poly(A)]–enriched mRNA as previously described (*14*). Libraries were polymerase chain reaction (PCR)–amplified for 9 to 14 cycles, size-selected using tris-boric acid-EDTA (TBE) gels or one-sided 0.8× AMPure cleanup, quantified by the Qubit dsDNA HS Assay Kit (Thermo Fisher Scientific), and 75-bp single-end–sequenced on a HiSeq 4000 or NextSeq 500 (Illumina).

### Cross-linking for ChIP-seq

For histone marks, PU.1, C/EBPβ, and RNA Pol II ChIP-seqs, culture media were removed and plates were washed once with PBS and then fixed for 10 min with 1% formaldehyde (Thermo Fisher Scientific) in PBS at room temperature; reaction was then quenched by adding glycine (Thermo Fisher Scientific) to 0.125 M. For STAT6, PPARγ, and EGR2 ChIP-seq, cells were cross-linked for 30 min with 2 mM DSG (Pierce) in PBS at room temperature. Subsequently, cells were fixed for 10 min with 1% formaldehyde at room temperature, and the reaction was quenched with 0.125 M glycine. After fixation, cells were washed once with cold PBS and then scraped into supernatant using a rubber policeman and pelleted for 5 min at 400*g* at 4°C. Cells were transferred to Eppendorf DNA LoBind tubes and pelleted at 700*g* for 5 min at 4°C, snap-frozen in liquid nitrogen, and stored at −80°C until ready for ChIP-seq protocol preparation.

### Chromatin immunoprecipitation

ChIP was performed in biological replicates as described previously (*44*). Samples were sonicated using a probe sonicator in 500-μl lysis buffer [10 mM tris-HCl (pH 7.5), 100 mM NaCl, 1 mM EDTA, 0.5 mM EGTA, 0.1% deoxycholate, 0.5% sarkozyl, and 1× protease inhibitor cocktail]. After sonication, 10% Triton X-100 was added to 1% final concentration, and lysates were spun at full speed for 10 min. One percent was taken as input DNA, and immunoprecipitation was carried out overnight with 20-μl Protein A Dynabeads (Invitrogen) and 2-μg specific antibodies for PU.1 (Santa Cruz Biotechnology, sc-352X), H3K4me2 (Millipore, 07-030), H3K4me3 (Millipore, 04-745), H3K27ac (Active Motif, 39135), RNA Pol II (GeneTex, GTX102535), STAT6 (Santa Cruz Biotechnology, sc-374021), EGR2 (Abcam, ab43020), and C/EBPβ (Santa Cruz Biotechnology, sc-150). Beads were washed three times each with wash buffer I (20 mM tris-HCl, 150 mM NaCl, 0.1% SDS, 1% Triton X-100, and 2 mM EDTA), wash buffer II (10 mM tris-HCl, 250 mM LiCl, 1% IGEPAL CA-630, 0.7% Na-deoxycholate, and 1 mM EDTA), tris-EDTA (TE) 0.2% Triton X-100, and TE 50 mM NaCl and subsequently resuspended 25-μl 10 mM tris-HCl (pH 8.0) and 0.05% Tween 20, and sequencing libraries were prepared on the Dynabeads as described below.

For PPARγ ChIP-seq, fixed cells were lysed in 500-μl radioimmunoprecipitation assay (RIPA) lysis buffer [20 mM tris-HCl (pH 7.5), 1 mM EDTA, 0.5 mM EGTA, 0.1% SDS, 0.4% Na-deoxycholate, 1% NP-40 alternative, 0.5 mM dithiothreitol (DTT), and 1× protease inhibitor cocktail (Sigma-Aldrich)] and chromatin was sheared using a probe sonicator. One percent was taken as input DNA, and immunoprecipitation was carried out overnight with 20-μl Protein A Dynabeads (Invitrogen) and 2 μg of both PPARγ antibodies (Santa Cruz Biotechnology, sc-271392 and sc-7273). Beads were then collected using a magnet and washed with 175-μl ice cold buffer as indicated by incubating samples on ice for 3 min: three times with RIPA wash buffer [20 mM tris-HCl (pH 7.5), 1 mM EDTA, 0.5 mM EGTA, 0.1% SDS, 0.4% Na-deoxycholate, 1% NP-40 alternative, 0.5 mM DTT, and 1× protease inhibitor cocktail (Sigma-Aldrich)], six times with LiCl wash buffer [10 mM tris-HCl (pH 7.5), 250 mM LiCl, 1 mM EDTA, 0.7% Na-deoxycholate, 1% NP-40 alternative, and 1× protease inhibitor cocktail (Sigma-Aldrich)], twice with TET [10 mM tris-HCl (pH 8.0), 1 mM EDTA, 0.2% Tween 20, and 1× protease inhibitor cocktail (Sigma-Aldrich)], and once with TE-NaCl [10 mM tris-HCl (pH 8.0), 0.1 mM EDTA, 50 mM NaCl, and 1× protease inhibitor cocktail (Sigma-Aldrich)]. Bead complexes were resuspended in 25-μl TT [10 mM tris-HCl (pH 8.0) and 0.05% Tween 20], and sequencing libraries were prepared on the Dynabeads as described below.

### ChIP-seq library preparation

ChIP libraries were prepared while bound to Dynabeads using a NEBNext Ultra II Library preparation kit (NEB) using half reactions. DNA was polished, poly(A)–tailed and ligated after which dual UDI (IDT) or single (Bioo Scientific) barcodes were ligated to it. Libraries were eluted and cross-links reversed by adding to the 46.5-μl NEB reaction, 16-μl water, 4-μl 10% SDS, 4.5-μl 5 M NaCl, 3-μl 0.5 M EDTA, 4-μl 0.2 M EGTA, 1-μl RNAse (10 mg/ml), and 1-μl proteinase K (20 mg/ml), followed by incubation at 55°C for 1 hour and 75°C for 30 min in a thermal cycler. Dynabeads were removed from the library using a magnet, and libraries were cleaned up by adding 2-μl SpeedBeads 3 EDAC (Thermo Fisher Scientific) in 124-μl 20% polyethylene glycol, molecular weight 8000/1.5 M NaCl, mixing well and then incubating at room temperature for 10 min. SpeedBeads were collected on a magnet and washed two times with 150-μl 80% ethanol for 30 s. Beads were collected, and ethanol was removed following each wash. After the second ethanol wash, beads were air-dried and DNA-eluted in 12.25-μl 10 mM tris-HCl (pH 8.0) and 0.05% Tween 20. DNA was amplified by PCR for 14 cycles in a 25-μl reaction volume using NEBNext Ultra II PCR master mix and 0.5 μM each Solexa 1GA and Solexa 1GB primers. Libraries were size-selected using TBE gels for 200 to 500 bp, and DNA was eluted using gel diffusion buffer [500 mM ammonium acetate (pH 8.0), 0.1% SDS, 1 mM EDTA, and 10 mM magnesium acetate] and purified using ChIP DNA Clean & Concentrator (Zymo Research). Sample concentrations were quantified by the Qubit dsDNA HS Assay Kit (Thermo Fisher Scientific) and 75-bp single-end–sequenced on HiSeq 4000 or NextSeq 500 (Illumina).

### ATAC-seq library preparation

Approximately 80K cells were lysed in 50-μl room temperature ATAC lysis buffer [10 mM tris-HCl, (pH 7.4), 10 mM NaCl, 3 mM MgCl_2_, and 0.1% IGEPAL CA-630], 2.5-μl DNA Tagmentation Enzyme mix (Nextera DNA Library Preparation Kit, Illumina) was added. The mixture was incubated at 37°C for 30 min and subsequently purified using the ChIP DNA purification kit (Zymo Research) as described by the manufacturer. DNA was amplified using the Nextera Primer Ad1 and a unique Ad2.n barcoding primer using NEBNext High-Fidelity 2× PCR MM for 8 to 14 cycles. PCRs were size-selected using TBE gels for 175 to 350 bp, and DNA was eluted using gel diffusion buffer [500 mM ammonium acetate (pH 8.0), 0.1% SDS, 1 mM EDTA, and 10 mM magnesium acetate] and purified using ChIP DNA Clean & Concentrator (Zymo Research). Samples were quantified by the Qubit dsDNA HS Assay Kit (Thermo Fisher Scientific) and 75-bp single-end–sequenced on HiSeq 4000 or NextSeq 500 (Illumina).

### H3K4me3 HiChIP

For H3K4me3 HiChIP, 10 million formaldehyde cross-linked cells per condition in biological replicates were used. HiChIP was performed as described before (*29*). In our experiments, 375 U of Mbo I (NEB, R0147M) restriction enzyme was used for chromatin digestion. Shearing was performed in three Covaris microtubes per sample and using the following parameters on a Covaris E220 (Fill Level = 6, Duty Cycle = 5, PIP = 140, Cycles/Burst = 200, Time = 200 s). H3K4me3 IP was performed using 7.5 μg of antibody (Millipore, 04-745). Final PCR was performed using NEBNext High-Fidelity PCR MM and Nextera general Primer Ad1 and specific Nextera Primer Ad2.n. PCR product was run on a TBE gel (Invitrogen), and libraries were size-selected from 250 to 700 bp and cleaned up using 150-μl gel diffusion buffer [500 mM ammonium acetate (pH 8.0), 0.1% SDS, 1 mM EDTA, and 10 mM magnesium acetate] and purified using ChIP DNA Clean & Concentrator (Zymo Research). Samples were quantified by the Qubit dsDNA HS Assay Kit (Thermo Fisher Scientific) and 75-bp paired-end–sequenced on a NextSeq 500 (Illumina).

### Data mapping

Custom genomes were generated for BALB, NOD, PWK, and SPRET mice from the C57 or mm10 genome as before (*14*) using MMARGE v1.0 (*45*) and the variant call format (VCF) files from the Mouse Genomes Project (*46*). Data generated from different mouse strains were first mapped to their respective genomes using STAR v2.5.3 (*47*) for RNA-seq data or bowtie2 v2.2.9 (*48*) for ATAC-seq, ChIP-seq, and HiChIP data. Then, the mapped data were shifted to the mm10 genome using the MMARGE v1.0 “shift” function (*45*) for downstream comparative analyses.

### RNA-seq data analysis

#### RNA-seq data processing

Transcripts were quantified using HOMER v4.11.1 “analyzeRepeats” script (*15*). Transcripts per kilobase million (TPM) values were reported by using the parameters -count exons -condenseGenes -tpm. Log-scaled TPM values were computed by log_2_(TPM + 1). Raw read counts within transcripts were reported by using the parameters -count exons -condenseGenes -noadj. Differentially expressed genes were identified by feeding raw read counts into DESeq2 (*49*) through the “getDiffExpression” script of HOMER. IL-4–induced and IL-4–repressed genes were called by fold changes greater than 2 or less than half, respectively, together with *q* values smaller than 0.05. Gene ontology analysis was performed using Metascape (*50*).

#### Categorization of strain-differential genes

Strain-differential genes were defined on the basis of pairwise comparisons between C57 and one of the other strains as being called IL-4–induced or IL-4–repressed in one strain but not in the other. Strain-differential IL-4–induced genes were further classified into three categories based on the relative level of basal expression between the induced strain versus the noninduced strain: high basal, equal basal, and low basal. In the high basal group, the noninduced strain has at least 1.5-fold greater basal expression level than the induced strain. The direction of difference flipped for the low basal group where the induced strain has over 1.5-fold greater basal expression than the noninduced strain. The genes in between are categorized into the equal basal group.

#### F1 mice data processing

RNA-seq data from F1 mice were mapped to both parental genomes (C57 and SPRET) and analyzed in the same way as before (*14*). In short, the read counts for each transcript were multiplied by the ratio of reads overlapping mutations times 10 and assigned to the parental genomes. Transcripts without any assigned reads in one of the F1 alleles were filtered out. To determine cis versus trans effects of genetic variation on gene expression, the difference of fold change between parental alleles and F1 alleles were calculated. The genes with majorly cis effects were defined by −1 < log_2_(parental fold change) – log_2_(F1 fold change) < 1, while those with majorly trans effects were defined by F1 fold change < parental fold change for genes with over ±2 fold-change in parental alleles.

### WGCNA analysis

For each strain, a differential gene expression analysis was performed to compare IL-4 to basal with Limma Voom (*51*). A linear model was fit for all five differential comparisons at once, and 1912 genes that were significant with *q* value below 0.05 and an absolute fold change of 1.5 in any comparison were included in a WGCNA (*52*). WGCNA was performed with a softpower value of 20, and a signed network was generated. Modules were cut with minimum module size of 50 and a cut height of 0.999 including PAM-stage. Nine modules were detected of which two genes were part of the gray (nonconnected) module that was subsequently excluded. Module Eigengenes (ME) were calculated and visualized using the verbose-boxplots function that also performed a Kruskal-Wallis significance test to test whether all ME values belong to the same distribution, and all modules were significantly different between conditions (all *P* values below <0.0012). Two modules exhibited consistent differential expression between IL-4 and notx across strains, while the other six modules were most prominently influenced in a strain-specific manner. Modules were annotated with Metascape (*50*).

### ATAC-seq and ChIP-seq data analysis

On the basis of the HOMER tag directories created from mapped sequencing data, the reproducible ATAC-seq and transcription factor ChIP-seq peaks were identified by using HOMER to call unfiltered 200-bp peaks (parameters -L 0 -C 0 -fdr 0.9 -size 200) and running IDR v2.0.3 on replicates of the same sample with the default parameters (*53*). The levels of histone modifications and RNA Pol II were quantified within ±500 bp around the centers of reproducible ATAC-seq peaks using HOMER annotatePeak.pl with parameters “-size -500,500 -norm 1e7.” The transcription factor binding intensities were quantified within ±300 bp around the identified ChIP-seq peaks using parameters “-size -150,150 -norm 1e7.” For comparisons across multiple samples (e.g., different time points, mouse strains, and transcription factors), we merged the set of peaks first using HOMER mergePeaks “-d given” before quantifying the features above. To visualize the average profile of a dataset around a certain set of peaks, we used HOMER annotatePeaks.pl with parameters “-norm 1e7 -size 4000 -hist 20” to help compute the histograms of 20-bp bins within ±2000 bp regions.

### Identification of IL-4–responsive regulatory elements

IL-4–responsive enhancers were identified by the strong fold changes of H3K27ac and RNA Pol II at intergenic or intronic open chromatin. Reproducible ATAC-seq peaks called from each mouse strain for the basal and IL-4 conditions were first merged and then annotated for genomic positions and the enrichment of H3K27ac and RNA Pol II within ±500 bp using HOMER v4.11.1. On the basis of the genomic annotations from HOMER annotatePeaks.pl, we classified regions at promoter-transcription start sites (TSS) as promoters and regions at intergenic or intronic positions as enhancers. Regions with less than 16 normalized tags of H3K27ac or less than 8 normalized tags of RNA Pol II were filtered out. For the remaining promoters and enhancers, we computed the fold changes of the normalized tags of H3K27ac and RNA Pol II between basal and IL-4 conditions for each mouse strain. Regions were called IL-4–induced or IL-4–repressed if there were at least 2.5-fold increases or decreases, respectively, from basal to IL-4 state for both histone markers. Regions with less than 1.4-fold changes were called neutral elements.

### Super enhancer

We used ROSE to call super enhancers for the five mouse strains (*25*). The active enhancers were first merged within each strain for both basal and IL-4 conditions to obtain a set of starting conventional enhancers. Then, the ROSE algorithm was run for each strain on the mapped H3K27ac ChIP-seq data with the parameter “-t 2500” to exclude TSS. The overall activity of a super enhancer was quantified by the H3K27ac ChIP-seq read counts within the entire identified super enhancer region.

### H3K4me3 HiChIP

#### H3K4me3 ChIP-seq HiChIP reference preprocessing

H3K4me3 ChIP-seqs from basal and 24-hour IL-4–stimulated macrophages were performed in duplicate with input controls. Fastq files were aligned with bowtie2 (*48*) to the mm10 reference genome, and peak calling was done with MACS (*54*) for each replicate separately. Significant peaks were merged using bedtools (*55*) into a general bed file that was used as corresponding peak file for MAPS.

#### H3K4me3 HiChIP preprocessing

HiChIP sequencing (HiChIP-seq) data were processed with MAPS (*56*) at 5000-bp resolution as described previously for proximity ligation-assisted ChIP-seq (PLAC-seq) (*28*) for all four samples separately, basal and 24-hour IL-4 duplicate samples combined, and a merge of all four samples.

#### Differential analysis

To identify interactions that were significantly stronger in Il-4 or control, a differential analysis was performed as described in (*28*). Briefly, significant interactions that were identified in the combined duplicate analysis of IL-4 and notx were merged in a general interaction set. Paired-end read counts that fell within these interactions were quantified for each sample separately. The quantified matrix of all significant interactions for all cell types was used as input for Limma (*57*) differential interaction analysis. A linear model was fit, with one pairwise contrast (IL-4 versus control), with and without batch correction. No interactions were identified that were significantly different between IL-4 and control by either method (false discovery rate < 0.1, and absolute log_2_ fold change > 1). Hence, the combined interaction set (generated using both IL-4 and control samples) was used for downstream analysis.

### Interactions among promoters and enhancers

Significant interactions captured by HiChIP-seq were overlapped with previously identified active promoters and enhancers for the five mouse strains using HOMER mergePeaks “-d 2500” to identify three categories of interactive pairs: enhancer-enhancer, enhancer-promoter, and promoter-promoter. Enhancer-promoter interactions have enhancers on one end and promoters on the other end, while enhancer-enhancer or promoter-promoter interactions are the linked pairs of enhancers or promoters, respectively. We ended up with 145,907 enhancer-enhancer interactions, 81,411 enhancer-promoter interactions, and 10,710 promoter-promoter interactions. To better understand the regulatory landscape associated with IL-4 stimulation, we subsequently focused on enhancer-promoter interactions that contained IL-4–induced, IL-4–repressed, and/or IL-4–neutral promoters on one end and IL-4–induced, IL-4–neutral, and/or IL-4–repressed enhancers on the other end, and quantified the number of interactions between these possible promoter-enhancer combinations in nine categories as a contingency table. Fisher’s exact test was applied to the contingency table to determine whether any of the categories were significantly different for three comparisons of interest: IL-4–induced enhancer/promoter interactions versus noninduced enhancer/promoters; IL-4–repressed enhancer/promoter interactions versus nonrepressed enhancer/promoters; and IL-4–induced enhancer/promoter interactions versus IL-4–repressed enhancer/promoter interactions. For enhancer-enhancer interactions, we preselected enhancers that have at least fourfold difference in H3K27ac ChIP-seq tags between any two strains under the 24-hour IL-4 condition to obtain a set of strongly strain-differential enhancers. We then computed the Pearson correlation of H3K27ac tags across the five strains for every pair of interactive enhancers among the preselected set. To obtain noninteractive enhancers, we either randomly paired preselected enhancers on the same chromosome (same-chromosome random enhancers) or looked for enhancers within certain distances but not connected based on our data (distance-matched random enhancers).

### Genetic variants at local and connected enhancers

Genetic variation between C57 and the other four strains at strain-differential enhancers was extracted using MMARGE annotate_mutations (*45*), which was based on the VCF files from the Mouse Genomes Project (*46*). Variants were searched within ±150 bp around the centers of enhancers. At least one genetic variant from the comparative strain needs to be present within the search area for such enhancer to be counted as having variants.

### Motif analysis

#### Motif enrichment analysis

Given a certain set of peaks, we used HOMER findMotifsGenome.pl with parameters “-size 200 -mask” to identify de novo motifs and their matched known motifs (*15*). The background sequences were either the default random sequences or a different set of peaks from a comparative condition in the main text and in the figure legends.

#### Motif mutation analysis

To integrate the genetic variation across mouse strains into motif analysis, we used MAGGIE, which is able to identify functional motifs out of the currently known motifs by testing for the association between motif mutations and the changes in specific epigenomic features (*32*). The known motifs are obtained from the JASPAR database (*58*). We applied this tool to strain-differential IL-4–responsive enhancers and transcription factor binding sites. Strain-differential IL-4–responsive enhancers were defined as previously described for KLA-responsive enhancers (*32*). In brief, from every pairwise comparison across the five strains, enhancers identified as “IL-4 activated” or “IL-4 repressed” only in one of the compared strains were called strain-differential and were pooled together. For enhancer sites to be included in the analysis, enhancer activity had to be differentially regulated between two strains. As required by MAGGIE, sequences from the genomes of the responsive strains were input as “positive sequences,” and those from the other strains as “negative sequences.” Strain-differential transcription factor binding sites were defined by reproducible ChIP-seq peaks called in one strain but not in the other. Positive sequences and negative sequences were specified as sequences from the bound and unbound strains, respectively. The output *P* values with signs indicating directional associations were averaged for clusters of motifs grouped by a maximum correlation of motif score differences larger than 0.6. Only motif clusters with at least one member showing a corresponding gene expression larger than 2 TPM in BMDMs were shown in figures.

### Categorization of IL-4–induced enhancers

Among the strain-differential IL-4–induced enhancers as described above, we further split them into three categories based on the level of H3K27ac under the basal condition in noninduced strains. High basal enhancers have more than twofold stronger H3K27ac in noninduced strains, while low basal enhancers have more than twofold stronger H3K27ac in induced strains (lower basal H3K27ac in noninduced strains). Equal basal enhancers are those in between.

### Deep learning

#### Neural network training

We adapted a similar strategy as AgentBind (*59*) for our training procedure. We started with a pretrained DeepSEA (*26*) model consisting of three convolutional layers and two fully connected layers and then fine-tuned it to generate three models based on our data: IL-4 active enhancers versus random backgrounds (auROC = 0.894), IL-4–induced enhancers versus random backgrounds (auROC = 0.919), and IL-4–induced enhancers versus noninduced enhancers (auROC = 0.796). The enhancer sequences were extended to 300 bp long. In all experiments, we left out sequences on chromosome 8 for cross-validation and sequences on chromosome 9 for testing. IL-4 active enhancers and noninduced enhancers were from C57 mice, while IL-4–induced enhancers were pooled from all the five strains to reach a comparable sample size. Random genomic backgrounds were generated by randomly selecting nearby guanine-cytosine (GC) content-matched equal-length sequences on the mm10 genome. We applied binary cross-entropy as the loss function. During each training, the initial learning rate was set as 1 × 10^−4^ and reduced by a factor of 0.9 when learning stagnated. The training process stopped when the loss value had not decreased for more than 20 epochs.

#### DeepLIFT and importance score

We used DeepLIFT (*27*) to generate importance scores with single-nucleotide resolution using uniform nucleotide backgrounds. For each input sequence, we generated two sets of scores, one for the original sequence and the other for its reverse complement. The final scores were the absolute maximum at each aligned position. We defined predicted functional nucleotides by the top 20% (i.e., top 60) positions within each input 300-bp sequence. To interpret the most important sequence patterns learned by neural networks, we computed the odds ratio of each 5-mer within top 10% of all 5-mers (*59*). Fisher’s exact test was performed to determine whether 5-mers were enriched. We used TOMTOM (*60*) to match 5-mers with known transcription factor binding motifs.

### Data and code availability

All sequencing data have been made available by deposition in the Gene Expression Omnibus (GEO) database: GSE159630. The UCSC genome browser was used to visualize sequencing data. The codes for neural network model training and interpretation are available on our Github repository: https://github.com/zeyang-shen/macrophage_IL4Response.

### Statistical analysis

Two independent groups were tested using Mann-Whitney *U* test for medians and using Levene’s test for variance. Gene expression comparisons were reported by adjusted *P* values (i.e., *q* values) from DESeq2 (*49*). Enrichment was computed by odds ratio and tested by Fisher’s exact test. Effect sizes were reported by Cohen’s *d*. All gene expression data are displayed as means with 95% confidence interval. All data distributions are shown with means, 25th percentiles, and 75th percentiles.

## SUPPLEMENTARY MATERIALS

Supplementary material for this article is available at http://advances.sciencemag.org/cgi/content/full/7/25/eabf9808/DC1

## Appendix

View/request a protocol for this paper from *Bio-protocol*.
